# A Curriculum on Digital Psychiatry for a US-Based Psychiatry Residency Training Program: Pilot Implementation Study

**DOI:** 10.2196/41573

**Published:** 2024-05-13

**Authors:** Sofia Noori, Siddharth Khasnavis, Eliza DeCroce-Movson, Morkeh Blay-Tofey, Evan Vitiello

**Affiliations:** 1 Yale Department of Psychiatry New Haven, CT United States; 2 Residency Training Program Yale Department of Psychiatry New York-Presbyterian Child and Adolescent Psychiatry New York, NY United States; 3 Department of Psychiatry Baylor Scott & White Health Temple, TX United States; 4 Department of Psychiatry University of North Carolina School of Medicine Chapel Hill, NC United States

**Keywords:** digital psychiatry, digital mental health, didactic curriculum, residency training, psychiatry residency, training classes, trainee response, residency curriculum, trainee feedback

## Abstract

**Background:**

Digital psychiatry, defined as the application of health technologies to the prevention, assessment, and treatment of mental health illnesses, is a growing field. Interest in the clinical use of these technologies continues to grow. However, psychiatric trainees receive limited or no formal education on the topic.

**Objective:**

This study aims to pilot a curriculum on digital psychiatry for a US-based psychiatry residency training program and examine the change in learner confidence regarding appraisal and clinical recommendation of digital mental health apps.

**Methods:**

Two 60-minute sessions were presented through a web-based platform to postgraduate year 2-4 residents training in psychiatry at a US-based adult psychiatry residency program. Learner confidence was assessed using pre- and postsession surveys.

**Results:**

Matched pre- and postsession quizzes showed improved confidence in multiple domains aligning with the course objectives. This included the structured appraisal of digital mental health apps (*P*=.03), assessment of a patient’s digital health literacy (*P*=.01), formal recommendation of digital health tools (*P*=.03), and prescription of digital therapeutics to patients (*P*=.03). Though an improvement from baseline, mean ratings for confidence did not exceed “somewhat comfortable” on any of the above measures.

**Conclusions:**

Our study shows the feasibility of implementing a digital psychiatry curriculum for residents in multiple levels of training. We also identified an opportunity to increase learner confidence in the appraisal and clinical use of digital mental health apps through the use of a formal curriculum.

## Introduction

Mental health disorders affect 1 in 3 people worldwide over a lifetime and account for nearly a third of all years lived with disability [1,2]. Advancement of technology has provided novel ways to expand care delivery, particularly in traditionally resource-poor settings. These changes are evident in the acceleration of remote and digital mental health services during the COVID-19 pandemic [3-5]. The growth of digital health technologies, in particular, provides a way to better allocate mental health resources [6].

Digital psychiatry is a term used to capture web-based and mobile media that provide mental health services. Digital health, more broadly, has garnered various definitions over the years, the first dating back to 2000 during the intersection of the internet, web-based services, and health care [7]. More recently, digital mental health was defined as “any application of digital health technology for mental health assessment, support, prevention, and treatment. This technological cluster includes mobile health (mHealth) applications, wearables, consumer neurotechnologies, virtual reality systems, online platforms, care coordination systems, assisted living ecosystems etc.” [8]. This approach to care delivery has seen tremendous growth. The first half of 2021 closed with US $14.7 billion invested across 372 US digital health deals with a US $39.6 million average deal size [9]. There are separately an estimated 10,000 patient-facing mental health apps in the consumer and research space and counting [10].

Amid the enthusiasm, the emphasis on digital mental health does raise new challenges for clinicians, including their ability to appraise technology for its accessibility, transparency of data collection, data sharing practices, and most importantly, its clinical value. [11,12]. Given that the emerging field of digital psychiatry will be crucial to the future of mental health care, it is imperative for future psychiatrists to be well-versed in the basics of digital psychiatry, its approaches, and its benefits and limitations to best guide patients. Unfortunately, exposure and training in this skill set within psychiatric residency training have not caught up with the growth of the larger field.

The value of information technology in psychiatry and the need for curriculum development in psychiatric residency programs was identified by Huang and Alessi [13]. Hariman et al [14] subsequently highlighted the need for a didactic curriculum to educate psychiatry trainees on the fundamentals of digital psychiatry. While a pilot curriculum was previously suggested, to our knowledge this has not been implemented in any training program to date [15]. Torous et al [16] have described and summarized evidence related to the growing field of digital psychiatry while highlighting that medical education programs fail to address this rapidly expanding overlap with clinical care. While educational approaches have emerged, these have focused on a singular individual, termed a “digital navigator,” rather than educating the general population of future clinicians [17,18]. Relatedly, an integrated training track in clinical informatics has been developed at a US psychiatry residency program, though it still does not address digital psychiatry or offer training across the residency program [19]. A recent survey among Italian learners including psychiatry trainees revealed that nearly 90% of respondents indicated a lack of discussion of digital psychiatry in their academic training, with the majority (54.4%) desiring training on the topic [20].

Didactics emerge as a natural partner for delivering training in digital psychiatry. In psychiatry residency, didactics provide a space for residents to learn the scientific foundations and clinical applications of modern psychiatric practice. At the Yale University School of Medicine’s Department of Psychiatry (Yale), a US-based psychiatry residency training program, didactic courses begin in the first year of residency and evolve into a half-day of protected time each week during postgraduate years (PGY) 2-4. Didactics take the form of lectures, observed interviews, team-based learning, problem-based learning, and case presentations. Most importantly, sessions are expected to reflect the most up-to-date literature on the biology, approach, and management of psychiatric pathologies. As such, didactic curricula are constantly changing to reflect modern practice. Programs have long been recognized for adapting their didactic curriculum and implementing new topics to better prepare trainees for careers after graduation [21-24]. Didactic sessions thus provide a means to address the gap in education on digital psychiatry.

This paper describes a curriculum implemented at a large psychiatry residency training program aimed at addressing the gap in trainee education on digital psychiatry. We discuss the feasibility of implementing the curriculum into didactic sessions in the residency program, the specifics of developing and implementing the curriculum, and review data capturing learner experience. Survey data specifically assess the change in learner confidence with regard to assessing and using digital therapeutics in future clinical encounters.

## Methods

### Digital Psychiatry Curriculum Design

The Digital Psychiatry Interest Group at Yale is a resident-led initiative led by second- through fourth-year psychiatry residents. Participation in the group has been open to trainees and faculty across departments. Historically, a diverse set of individuals including residents, fellows, and rotating medical students have been involved in the group. The group’s priority has been to increase awareness of digital mental health tools and their application in clinical care at the institution. This directive has aligned with observed gaps in residency didactics and the group’s organization and approach made it feasible to create an opportunity to implement a digital psychiatry training program. A structured digital psychiatry curriculum was thus undertaken by our group with several expectations for future residents including the ability to (1) define digital mental health (DMH) and common categories of DMH tools, (2) survey a patient’s digital health preparedness, (3) recommend DMH tools in a clinically useful manner, and (4) understand the elements of formally prescribing DMH apps. The purpose of the digital psychiatry curriculum was to expose trainees to a constantly expanding field of psychiatry through teachings and active learning didactics. This curriculum was to provide a background of knowledge that could be built upon through experiential learning so that residents could be better equipped to evaluate and recommend digital interventions in their clinical practice.

It was felt that a dedicated didactic curriculum for DMH was needed, rather than trusting that the rest of the residency training program would provide adequate exposure, for several reasons. As previously discussed, digital psychiatry is a continually growing and evolving field. Focus groups with the Digital Psychiatry Interest Group involving clinical attendings revealed that there was a gap in knowledge on the subject and wide variability in how mental health apps and digital therapeutics were incorporated into clinical training. By creating a dedicated curriculum, residents could have more standardized education on the topic which they could build further through future clinical encounters. Additionally, given the turnover in interest groups as students, residents, and fellows progress through their training and graduation, a recurring curriculum in the didactic series helped to solidify that the topic would continue to be covered throughout staffing changes. Last, having a standing curriculum required educators who were committed to staying updated on the latest developments in a rapidly evolving field.

Pre- and postsession surveys were developed through focus groups with faculty members who had experience in survey methods. The purpose of the surveys was to assess the current landscape of residents incorporating apps and digital therapeutics into their clinical practice, as well as to assess the impact the didactic sessions had on residents’ outlook and comfort in navigating patient encounters that might incorporate these technologies. The target population of the intervention and survey were PGY2 and PGY4 resident learners.

The curriculum was developed as 2 sessions which could be delivered in consecutive hours or weekly sessions. Each session incorporated didactic and experiential components. Six educations developed the curriculum, which allowed for those individuals to split presenting between the 2 sessions and promote discussions during small group breakout sessions. These educators were drawn from the Digital Psychiatry Interest Group, consisting of a range of trainees from junior residents to fellows.

Session 1 focused on an introduction to digital psychiatry and developing skills to assess new apps and technologies. It reviewed terminology relevant to DMH and challenges in the adoption of these apps and technologies in mental health. The session defined basic terms in the field and reviewed the current state of technology, including how these innovations are created, how insurers and regulatory bodies approach digital therapeutics and interventions, and the role of psychiatrists in them. For experiential learning, trainees learned about the American Psychiatric Association app evaluation model and used the schema to assess mental health apps and additionally the tool itself [25]. This app advisor framework was instrumental in building upon further active learning during the next didactic session. Session 1 concluded by exposing learners to leaders in the field of DMH, helping to show residents potential career paths in clinical, research, and industry settings. Additionally, local initiatives at their institution were highlighted to promote further involvement during their residency training experience.

In session 2, participants shifted their attention to patient readiness and practical aspects of prescribing or recommending apps. Learners were exposed to a framework on how to assess a patient’s digital health preparedness. Building upon that framework, trainees engaged in active learning through mock patient interviews using small breakout groups and focused on identifying barriers and cultivating a language for the recommendation of digital apps. The session also presented the formal prescription of apps using currently approved products, such as digital therapeutics, as examples. The differences between recommending an app and prescribing a digital therapeutic were highlighted, with recommendations on language and sample scripts that could be used when speaking with patients. Further regulatory information, such as how the Food and Drug Administration approves digital therapeutics, was reviewed. Learners then broke into small groups with a role-playing exercise to work through an example of digital therapeutic and the prescribing versus recommending distinction. Examples of apps that learners evaluated included Sleepio (Big Health), Somryst (Nox Health), reSET-O (Digital Therapeutics Alliance), EndeavorRx (Akili Interactive Labs), CBT-I Coach through the US Department of Veterans Affairs, Headspace (Headspace Health), Calm (Calm.com), Crisis Text Line (Crisis Text Line, Inc), and NOCD (NOCD Inc). These were chosen as they span a wide range of indications, industry, popularity, Food and Drug Administration approval, and cost.

In this pilot study, learners completed the sessions using a video teleconferencing platform due to changes in didactic structure amid the COVID-19 pandemic. The curriculum was designed to offer flexibility for face-to-face or web-based presentations and ultimately prioritizes experiential learning.

### Ethical Considerations

The proposed survey and data collection was granted an exemption by Yale University’s human research ethics institutional review board (IRB ID: 2000030130).

### Analysis

Residents participated in brief pre- and postsession questionnaires for both classes. A unique, nontraceable identifier was used to allow anonymity in the submissions. Surveys included ratings of confidence corresponding to skills reviewed in each session and self-reported frequencies of app prescribing. Trainees also provided feedback in an open-ended format and by rating the quality of instruction on a Likert scale.

Data were analyzed in Excel (Microsoft Corp) using qualitative statistics in order to assess the frequency of responses for grouped or themed items. Pre- and postsession survey questions in the format of a Likert scale that queried for comfort level in the skills taught during the sessions were analyzed using paired *t* tests to compare responses for identical questions before and after the curriculum implementation.

## Results

The data indicate a statistically significant improvement in resident comfort with all stated competencies of the curriculum. Namely, residents were significantly more comfortable in conducting a structured assessment of a digital mental health app for its clinical utility, were significantly more comfortable in assessing a patient’s digital health literacy, significantly more comfortable in formally recommending a digital health tool to patients, and significantly more comfortable prescribing a digital therapeutic to their patients. While the implementation of the digital psychiatry curriculum was a new intervention, and thus admittedly has a low number of participants which limits interpretation and generalizability of the results, all identified learning objectives of this curriculum were associated with measured improvement in learners’ comfort using these tools for clinical care.

However, average resident comfort did not exceed “somewhat comfortable” in any objective, possibly indicating that the curriculum could be further refined to complete these objectives, or that residents may benefit from further training or exposure to digital psychiatry competencies in the future. In qualitative feedback, residents commented that the role-playing exercises were especially helpful in learning to prescribe and recommend apps. Thus, future iterations of the curriculum could focus on more hypothetical case-based active learning. Some felt that the American Psychiatric Association app evaluation model was too lengthy to use in clinical practice and that a database of app evaluations from a reliable clinical organization may be more clinically useful, offering some insights into potential future projects by groups that are working to create resources in this space. Please refer to Tables 1 and 2 for the statistical analyses conducted in sessions 1 and 2, respectively.

**Table 1:** Session 1 data from corresponding pre- and postsession questionnaires.

Survey question	Presession (n=21)	Postsession (n=12)	*P* values (n=12)
How comfortable do you feel in conducting a structured assessment of a digital mental health application for its clinical utility? (1= not at all comfortable, 5= very comfortable), mean	1.8	2.5	.03
What are the barriers in your use of digital mental health resources in patient care? Select all that apply.	Unsure of which apps to recommend: 90.4% (n=19) Lack of evidence base: 42.9% (n=9) Patient’s level of interest: 38.1% (n=8) Cost to patient: 38.1% (n=8) Patient’s digital health literacy: 33.3% (n=7) Lack of time: 19% (n=4)	Not asked	N/A^a^
How often have you formally recommended the use of a digital mental health application with your patient in the past 6 months?	More than 5 times: 9.5% (n=2) 3-4 times: 4.7% (n=1) 1-2 times: 38.1% (n=8) Never: 47.7% (n=10)	Not asked	N/A

^a^N/A: Not applicable.

**Table 2:** Session 2 data from corresponding pre- and postsession questionnaires.

Survey question	Presession (n=16)	Postsession (n=13)	*P* values (n=9)
How comfortable do you feel in assessing a patient’s digital health literacy? (1= not at all comfortable, 5= very comfortable), mean	2.0	3.1	.01
How comfortable do you feel formally recommending a digital health tool to your patients? (1= not at all comfortable, 5= very comfortable), mean	1.9	2.8	.03
How comfortable do you feel prescribing a digital therapeutic to your patients? (1= not at all comfortable, 5= very comfortable), mean	1.8	2.8	.03
The content of this session will be helpful in my clinical practice (1= strongly disagree, 5= strongly agree), mean	Not asked	3.4	N/A^a^

^a^N/A: Not applicable.

Data from this project reveal that psychiatry trainees have started incorporating digital psychiatry in their clinical practice. In fact, 11 of 21 (52%) participants have recommended an app to a patient, with 2 of 21 (10%) participants recommending a digital mental health app more than 5 times to patients. However, there remain major gaps in that 19 of 21 (~91%) respondents were unsure of which apps to recommend, with nearly 9 of 21 (43%) participants raising concerns if these apps are supported by evidence. This supports the notion that there is a rapidly growing number of apps that can be overwhelming for the clinician to navigate in clinical encounters.

## Discussion

### Principal Results

This pilot study shows that a digital psychiatry curriculum can be implemented and incorporated into regular residency didactics, with meaningful growth in learner education through just a few interactive sessions. Residents showed significant improvement in their comfort level in terms of navigating how newer technologies, including digital therapeutics and mobile apps, intersect with clinical care. While the intersection of information technology and psychiatry has been apparent for decades, the field of digital psychiatry has received heightened attention recently due to the pressing need for virtual interventions from the COVID-19 pandemic. Although digital psychiatry is a rapidly growing field, very little is taught about how to evaluate or recommend digital tools to patients, or how to assess which patients can benefit from those tools. To our knowledge, this curriculum is the first residency-wide curriculum of any psychiatric residency training program in the United States. Our team sought to equip trainees with the knowledge and skills needed to safely evaluate and recommend apps to patients, and the critical evaluation of innovation that may drive the field forward.

Psychiatry residents are exposed to a number of didactics throughout their 4 years of training and these didactics are expected to adapt as the field shifts. While trainees may receive exposure to digital mental health apps and therapeutics during clinical encounters, these experiences vary depending on local interest and supervision. A dedicated curriculum helps standardize the trainee experience, particularly when structured with discussion and interactive sessions.

Our survey data confirms the value and demand for such education. Over 50% of our participants have recommended a digital mental health intervention to their patients. Yet, the vast majority (19/21, 90.4%) observed that a lack of knowledge regarding the appraisal of digital apps limited their use in clinical settings. That 90.4% (n=19) number is consistent with Italian survey data from a larger respondent pool outlining that learners, including psychiatry trainees, lack education on digital psychiatry. This underscores the gap between clinical need and education regarding the use of digital interventions. Our experience shows that even a brief didactic curriculum of 2 sessions increased learners’ comfort in identified competencies, including assessing mental health apps for their clinical utility, assessing a patient’s digital health literacy, and either recommending or formally prescribing digital interventions.

### Limitations

We also recognize several limitations in our report. First, our model was only implemented at a single academic institution. We hope that our encouraging findings serve as a rubric for others who are seeking to incorporate digital psychiatry education in residency training. This may allow for broader implementation and opportunities to report on training conducted at multiple sites and across a larger sample of residents. Likewise, our pilot implementation was limited to PGY2 and PGY4 residents based on scheduling constraints. We are eager to distribute the curriculum more widely for future cycles and additionally, tailor the experience based on residency year. An additional limitation of a pilot study was the inability to assess change in behavior or real-world outcomes with regard to digital therapeutic use in the clinical setting. This serves as another goal as our curriculum becomes solidified into future resident didactics.

### Comparison With Prior Work

This pilot study is intended to serve as a blueprint for other institutions that recognize the growing field of digital psychiatry and its importance to be incorporated into resident didactics to train future clinicians dedicated to mental health care. We hope to show that meaningful growth in learners’ comfort surrounding digital therapeutics can be accomplished with as little as 2 sessions. Further, we show consistency with a US sample of psychiatry residents compared with an Italian group of trainees in that 90.4% (n=19) of respondents noted a gap in their education related to concepts of digital psychiatry.

### Conclusions

In concluding our report, we highlight several observations that could be helpful to other educators and curriculum planners. First, faculty interest and familiarity with digital therapeutics may not be assured across institutions and therefore, early engagement with local experts is vital. This is particularly relevant in a rapidly changing field such as digital psychiatry. Creative strategies may include identifying nonclinical faculty with expertise in this subject or experts from subspecialties outside psychiatry. In our experience, for instance, an established Digital Psychiatry Interest Group and mentorship with both local and regional faculty helped to nucleate curriculum development.

Finally, future studies could examine if increased education about digital psychiatry impacts prescribing behaviors in trainees. While our survey data reveal increased resident comfort related to assessing patient digital health literacy, mental health apps, and digital therapeutics, we were unable to understand learners’ future behaviors in clinical encounters. A longitudinal study comparing these behaviors to another institution that does not have a comparable didactic curriculum could prove useful.

In conclusion, digital psychiatry is here to stay. Psychiatry trainees are incorporating digital mental health apps in their patient encounters; however, there is no established didactic curriculum to educate residents in this realm. We illustrated that it was feasible to implement a digital psychiatry curriculum into psychiatry residency didactics. We present one such didactic program in digital psychiatry established at Yale.

## Abbreviations

DMH: Digital Mental Health
mHealth: mobile health
PGY: postgraduate year

## Data Availability

The data sets generated or analyzed during this study are available from the corresponding author on reasonable request.
